# Prediction of delayed graft function and long-term graft survival by serum and urinary neutrophil gelatinase–associated lipocalin during the early postoperative phase after kidney transplantation

**DOI:** 10.1371/journal.pone.0189932

**Published:** 2018-01-05

**Authors:** Herbert Thomas Maier, Muhammad Imtiaz Ashraf, Christian Denecke, Sascha Weiss, Florian Augustin, Franka Messner, Natalie Vallant, Matthias Böcklein, Christian Margreiter, Georg Göbel, Johann Pratschke, Dietmar Öfner-Velano, Felix Aigner

**Affiliations:** 1 Department of Visceral, Transplant and Thoracic Surgery, Innsbruck Medical University, Innsbruck, Austria; 2 Daniel-Swarovski-Research Laboratory, Innsbruck Medical University, Innsbruck, Austria; 3 Charité Universitätsmedizin Berlin, Department of Surgery, Campus Virchow-Klinikum and Campus Mitte, Berlin, Germany; 4 Department of Medical Statistics, Informatics and Health Economics, Innsbruck Medical University, Innsbruck, Austria; Medizinische Universitat Graz, AUSTRIA

## Abstract

Neutrophil gelatinase-associated lipocalin (NGAL) has emerged as an early marker protein for kidney dysfunction in various clinical settings. In this prospective study we evaluated serial changes of serum and urinary NGAL within the first 7 days after kidney transplantation in 170 consecutive recipients. The main focus of this study was to assess the performance of serum and urinary NGAL in the prediction of delayed graft function (DGF) and two-year graft and patient survival. Serum and urine samples of 170 patients undergoing primary kidney transplantation from October 2010 to December 2012 were prospectively collected from day 0 to 7. NGAL was analyzed by ELISA. Multivariate regression models, receiver-operating characteristics (ROC), and areas under ROC curves (AUC) were used to identify predictors of DGF. DGF occurred in 52 patients (30.6%). Serum (AUC = 0.869) and urinary NGAL (AUC = 0.872) on postoperative day (POD) 2 could accurately predict DGF compared to serum creatinine (AUC = 0.619). Multivariate analyses revealed donor age, serum and urinary NGAL significantly associated with DGF (p<0.001). Recipient age was the only significant factor in a cox regression model influencing two-year graft and patient survival. In conclusion, serum and urinary NGAL are early predictors of DGF after kidney transplantation.

## Introduction

Early allograft dysfunction as well as long-term graft loss still represent major obstacles to successful organ transplantation. Besides immunological reactions due to donor and recipient incompatibility, ischemia-reperfusion injury (IRI) of the transplanted organ is the major process negatively affecting allograft function early after transplantation and long-term graft survival. Thus it is important to find new biomarkers that will rapidly and reliably detect acute and chronic allograft rejection as well as delayed graft function (DGF) [1]. Among a series of novel markers identified by high-throughput expression analyses was NGAL, a small member of the lipocalin family of lipophilic substrate binding proteins, that was found to be dramatically up-regulated in transplanted hearts immediately after transplantation [2]. NGAL was first described by Kjeldsen and Cowland as a protein associated with human neutrophil gelatinase [3]. Later, NGAL was also found to be synthesized in large amounts in the course of kidney injury where it was explored as a marker for compromised organ function [4,5].

DGF is a common complication after kidney transplantation, especially with increasing numbers of suboptimal donors, such as expanded criteria donors. It is associated with decreased short- and long-term graft survival rates [6].

DGF is usually diagnosed by need for dialysis, diuresis and plasma creatinine. However, plasma creatinine is affected by other factors such as body weight, age, sex and muscle metabolism [7].

Moreover, the detectable rise in serum creatinine occurs at a stage where a significant damage has already happened to the graft as late indicator of injury and rejection and recovery becomes challenging [8]. This signifies the need of an accurate and early biomarker of graft injury and rejection.

NGAL was shown to be an accurate biomarker of kidney injury in various experimental and clinical settings [9]. Hall et al [10] could demonstrate that NGAL, measured on the first postoperative day, could predict the need for dialysis in the first week after transplantation whereas serum creatinine could not. Likewise, significantly higher NGAL levels were found in patients immediately after kidney transplantation that developed acute rejection [11].

Recent studies could more or less confirm these results by analysing NGAL after kidney transplantation from expanded criteria donors [12, 13]. Cantaluppi et al. even showed tacrolimus-induced increase of serum NGAL suggesting a role as marker of drug toxicity [13].

The aim of our study was to examine serial serum and urinary NGAL expression in the early postoperative phase after kidney transplantation and to verify the significance of NGAL in predicting DGF and acute allograft rejection.

## Material and methods

### Patient selection

170 consecutive patients with end-stage renal disease undergoing kidney transplantation from October 2010 to December 2012 were prospectively enrolled after signed informed consent at our institution. Exclusion criteria were age less than 18 years, retransplantation and combined transplantation with another organ. None of the transplant donors were from a vulnerable population and all donors or next of kin provided written informed consent that was freely given. The study was approved by the Institutional Review Board (UN3710; Ethics Committee, Innsbruck Medical University).

### Sample collection and measurement of laboratory parameters

Serum and urine samples of 170 patients undergoing primary kidney transplantation were collected preoperatively and postoperatively from day one to 7 or discharge from hospital respectively. Preoperative samples were taken prior to surgery. Postoperative samples were taken during routinely performed blood collection in the morning. This was also the case with samples on postoperative day 1, these samples were not taken immediately after surgery. In six patients it was not possible to obtain urine samples on postoperative day 1 and in two patients on postoperative day 2 because of anuria. Urine collection was done via urinary catheter and morning urine after catheter removal, respectively. Serum and urinary NGAL levels were measured by ELISA (Quantikine, R&D Systems, MN, USA). Serum creatinine was measured by Jaffe method (Roche Diagnostics). Graft ultrasound was performed routinely and biopsies of the grafts were only performed on clinical assumption of graft rejection.

### Statistics

Absolute and relative frequencies are presented for nominal variables. To evaluate prospective diagnostic power receiver operating characteristic (ROC) curves including AUC data were generated at 48 hours after kidney transplantation. We used the CHI^2^-Test to compare categorical variables. T-test were applied to test parametric variables between groups and Mann-Whitney U tests in the non-parametric case, respectively. Multivariate analyses were performed by using a logistic regression model and by establishing a Cox proportional hazards model for time-to-event analyses. For all analyses, a p-value <0.05 was defined as statistically significant. Confidence intervalls (CI) were calculated on a 95% level. Statistical analyses were performed using the SPSS version 24.0 for Windows.

## Results

Our study sample included 170 recipients. Sociodemographical data and baseline characteristics are shown in Table 1. Fifty-two patients developed delayed graft function which was defined by the need for dialysis within the first week after transplantation according to the United Network for Organ Sharing [14]. In the DGF group, recipients and donors were older and ischemia time was longer, these data were significantly different. Charlson comorbidity index, duration and type of preoperative dialysis were comparable in both groups. In four patients acute rejection episodes were biopsy-proven (Patient #2: acute humoral rejection BANFF I focal low positive for C4d; Patient #32: cellular rejection BANFF IA focal low positive for HLA DR and C4d negative; Patient #57: acute cellular rejection BANFF IIB c4d negative; Patient #158: acute humoral rejection BANFF II c4d negative). None of the four patients developed donor specific antibodies. 165 patients received a calcineurininhibitor and mycophenolatmofetil-based immunosuppression therapy with or without induction therapy with basiliximab. Because of reasons of medical history and AB0 incompatible living donation five patients received immunosuppression as follows: Patient #33: Basiliximab, calcineurine inhibitor (CNI), Azathioprine (AZA), Methylprednisolon (MP); Patient #52: Campath, CNI, Mycophenolatmofetil (MMF), MP; Patient #92: Basiliximab, MMF, MP; Patient #140: Rituximab, immunoglobulin apheresis, CNI, MMF, MP; Patient #143: anti-thymoglobin, CNI, MMF, MP). Twenty patients underwent living donor kidney transplantation.

**Table 1:** Sociodemographical data of 170 kidney recipients and donors.

	Overall	Primary function	DGF	p
	N = 170	N = 118	N = 52	
*Patient baseline/preoperative data*				
Recipient sex (N—female)	61 (36%)	44 (37%)	17 (33%)	0.6
Recipient age (Mean / SD)	55 (14)	54 (14)	59 (12)	0.02
Donor age (Mean / SD)	54 (16)	52 (15)	60 (15)	<0.001
HLA missmatches (Mean / SD)	3 (1.5)	3 (1.6)	4 (1.3)	0.08
Ischemia time (minutes, Mean / SD)	763 (329)	716 (347)	867 (258)	0.002
Charlson Comorbidity Index (Mean / SD)	2.8 (1.2)	2.7 (1.1)	3.1 (1.5)	0.1
Type of Dialysis (N—HD)	113 (67%)	74 (74%)	39 (85%)	0.2
Duration of Dialysis (months, mean / SD)	45 (28)	42 (30)	51 (24)	0.09
Diuresis pretransplant (N—yes)	100 (59%)	71 (69%)	29 (62%)	0.4
Diuresis pretransplant (lt, median / IQR)	0.9 (1.5)	1.0 (1.7)	0.7 (1.2)	0.09
Living donation	20 (12%)	20 (17%)	0	
*Clinical outcome*				
Episode of acute rejection	4 (2%)	0	4 (8%)	
Graft loss after 1 year	14 (8%)	6 (5%)	8 (15%)	
Death after 1 year	9 (5%)	5 (4%)	4 (8%)	
Graft loss after 2 years	19 (11%)	7 (6%)	12 (23%)	
Death after 2 years	11 (7%)	5 (4%)	6 (12%)	
Graft loss after 5 years	30 (18%)	14 (12%)	16 (31%)	
Death after 5 years	15 (9%)	8 (7%)	7 (14%)	
*initial immonosuppression*				
CNI, MP, MMF	37 (22%)	31 (26%)	6 (12%)	
Basiliximab, CNI, MP, MMF	128 (75%)	83 (70%)	45 (87%)	
other	5 (3%)	4 (3%)	1 (2%)	

DGF: delayed graft function; SD: standard deviation; CNI: calcineurine inhibitors; MP: Methylprednisolon; MMF: Mycophenolatmofetil; IQR: interquartile range; HD: Haemodialysis

### Postoperative creatinine, urine output and serum and urinary NGAL levels

To demonstrate differences in the expression profiles of typical surrogate parameters of kidney function and NGAL, serum and urinary NGAL levels, serum creatinine and urine output was measured from postoperative day 1 to 7. Recipients were divided into two groups (DGF versus primary function). Table 2 shows median values of serum und urinary NGAL, serum creatinine and urine output levels from postoperative day 1 to 7 in both groups. To avoid bias, data are also shown without living donation kidney transplantations as DGF occurs rarely in these patients. Expectedly, all surrogate parameters were higher in the DGF group compared to patients with primary function.

**Table 2:** Creatinine, urinary NGAL, serum NGAL and urinary output levels from postoperative days 1–7.

**creatinine mg/dl (median/IQR)**	**d1**	**d2**	**d3**	**d4**	**d5**	**d6**	**d7**
overall	6,6 [4,9–8,8]	5,1 [3,7–6,6]	4,2 [3–5,9]	3,6 [2,2–5]	2,8 [1,7–4,6]	2,2 [1,5–4,3]	2 [1,4–3,5]
primary function overall	6,5 [4,7–8,8]	4,6 [3,5–6,4]	3,7 [2,8–4,9]	2,6 [1,8–3,9]	2,1 [1,5–3]	1,7 [1,3–2,3]	1,5 [1,2–2,1]
primary function without living donation	6,5 [4,6–8,9]	4,6 [3,7–6,1]	3,7 [2,8–4,9]	2,7 [1,8–3,9]	2,1 [1,5–3]	1,7 [1,3–2,6]	1,6 [1,1–2,2]
DGF	6,8 [5,6–9,1]	5,6 [4,2–7,1]	5,8 [4,6–7,3]	5,4 [4,4–7,3]	5,4 [4,3–7,5]	5,3 [3,7–6,6]	4,5 [3,4–5,8]
**serum NGAL ng/ml (median/IQR)**	**d1**	**d2**	**d3**	**d4**	**d5**	**d6**	**d7**
overall	255,4 [189,8–295,5]	195,5 [162,1–249,4]	177,6 [140,9–222,4]	151,3 [121,3–206,1]	140,6 [114,5–187,8]	133 [112,5–189,6]	134 [112,3–184,8]
primary function	233,3 [186,8–278,6]	176,6 [145,7–210,6]	156,1 [131,4–186,6]	132,6 [115,4–162,8]	127,5 [106,4–143,6]	120,8 [101,4–136,5]	119,8 [101,6–140,9]
primary function without living donation	243,6 [188,3–286,2]	178,6 [147,0–220,1]	156,6 [131,4–186,5]	132,6 [116,4–162,8]	125,2 [107,4–143,6]	120,5 [100,1–134,7]	119,8 [102,6–140,9]
DGF	298,9 [229,1–316,9]	277,8 [216,4–298,3]	250,6 [206,8–284,4]	248,8 [200,5–276,3]	204,2 [183,7–244]	212,5 [186,4–243,8]	197,3 [176,5–247,6]
**urinary NGAL ng/ml (median/IQR)**	**d1**	**d2**	**d3**	**d4**	**d5**	**d6**	**d7**
overall	147,7 [98,3–247,4]	79,2 [35,6–171,9]	39 [19,5–128,5]	29 [15,7–99,5]	22 [12,6–57,9]	17,3 [9,6–45,8]	15,3 [8,7–49,2]
primary function	126,6 [89,7–187,2]	54,5 [26,6–101,9]	25,8 [15,3–49]	21,5 [12,9–31,7]	18 [10,5–30]	12,6 [8,5–22,4]	12,7 [7–18,6]
primary function without living donation	126,6 [87,8–187,2]	54,5 [25,7–105,0]	26,3 [15,9–55,2]	21,4 [12,8–32,1]	17,9 [11,7–30,4]	12,6 [8,5–22,2]	12,6 [6,7–18,6]
DGF	239,4 [187,1–317,7]	189,3 [133–297,9]	177,1 [109,3–270,1]	144,9 [56,1–188,3]	130,2 [40,5–185,3]	121,7 [37,5–176,9]	116,4 [32,4–155,9]
**urine output liter (median/IQR)**	**d1**	**d2**	**d3**	**d4**	**d5**	**d6**	**d7**
overall	2,23 [1,275–3,48]	2,72 [1,5–3,75]	2,63 [1,825–3,525]	2,52 [1,65–3,275]	2,58 [1,8–3,255]	2,46 [1,88–3,22]	2,6 [2,06–3,205]
primary function	2,8 [1,97–3,9]	3,06 [2,58–4,15]	2,93 [2,35–3,85]	2,75 [2,15–3,62]	2,87 [2,3–3,53]	2,8 [2,23–3,34]	2,85 [2,2–3,4]
primary function without living donation	2,67 [1,77–3,77]	2,96 [2,44–4,13]	2,92 [2,42–3,79]	2,75 [2,158–3,6]	2,86 [2,32–3,528]	2,82 [2,208–3,348]	2,84 [2,225–3,33]
DGF	0,795 [0,19–1,778]	0,72 [0,28–1,855]	1,220 [0,33–2,170]	1,41 [0,408–2,393]	1,485 [0,658–2,165]	1,815 [0,613–2,4]	2,175 [0,69–2,863]

DGF: delayed graft function; IQR: interquartile range

### Prediction of DGF by serum and urinary NGAL

**Fig 1:**
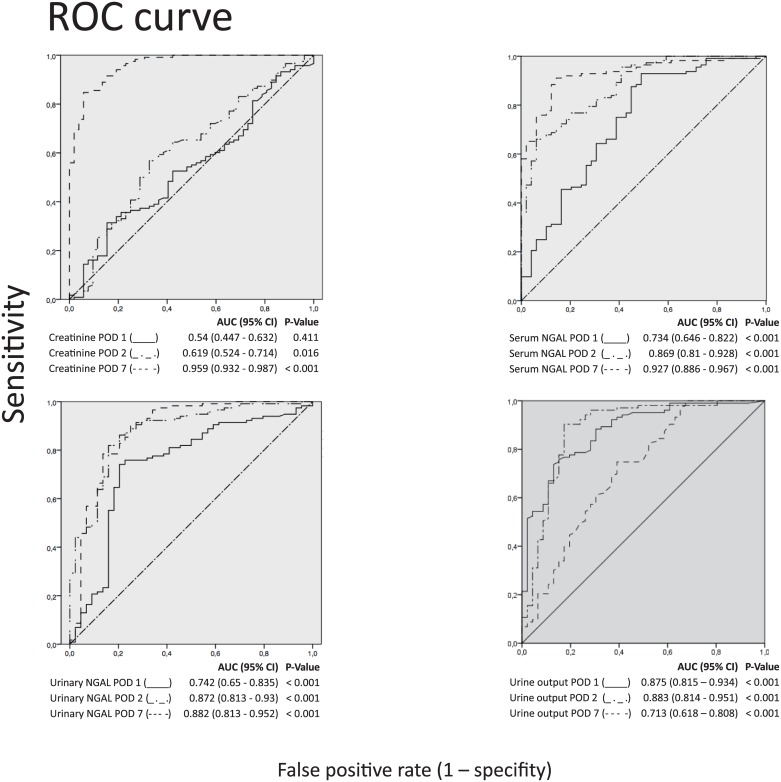
Receiver-operating characteristic (ROC) curves for urine output, serum NGAL, urinary NGAL and serum creatinine at posttransplant day 1, 2 and 7 (POD 1,2,7) for predicting delayed graft function.

To assess the predictive value of serum and urinary NGAL to detect DGF receiver operating characteristic (ROC) analyses was performed. Regarding the area under the ROC curve (AUC), urinary and serum NGAL could predict DGF highly accurate at second postoperative day compared to serum creatinine (Fig 1). The AUC was highest for urinary NGAL (0.872) followed by serum NGAL (0.869). Higher AUC levels of urinary and serum NGAL on POD 2 compared to POD 1 demonstrate the prognostic value of prediction of DGF at this time point. Predictable power of NGAL could be demonstrated for both urine and serum NGAL as early as POD 1. Likewise, the AUC for urine output is highest on POD 2 with highly accurate prediction of DGF (0.883). Interestingly, compared to creatinine, serum and urinary NGAL, the AUC decreases on POD 7 (0.713) regarding urine output.

### Association of NGAL and DGF, graft and patient survival

To test independent parameters for DGF with regard to NGAL expression, multivariate analyses were performed. Multivariate analyses including clinically relevant factors (recipient and donor age, cold ischemia time, HLA mismatch) revealed that serum and urinary NGAL were independent predictors of DGF on POD 2 compared to serum creatinine at this time point (Table 3). Furthermore, urine output at this time point remained independently associated with DGF. Data are also shown without living donation kidney transplantation with similar results. Cox regression model for graft loss and patient death (with or without functioning graft) two years and five years after transplantation (Table 4) identified recipient age as the only independent prognostic factor for patient loss. Two years after transplantation, recipient age is also an independent prognostic factor for graft loss.

**Table 3:** Univariate and multivariate analyses to identify predictors of DGF.

Variable	univariate analysis		multivariate analysis		univariate analysis without living donation		multivariate analysis without living donation	
	OR (95% CI)	*P*	HR (95% CI)	*P*	OR (95% CI)	*P*	HR (95% CI)	*P*
**Sex f>m**	1.2 [0.6–2.4]	0.6			1.3 [0.6–2.6]	0.5		
**recipient age (per 10 years)**	1.4 [1.0–1.8]	**0.02**	0.9 [0.5–1.6]	0.6	1.2 [0.9–1.6]	0.1	0.8 [0.3–2.0]	0.6
**donor age (per 10 years)**	1.5 [1.2–2]	**0.002**	1.7 [0.9–3.1]	0.1	1.5 [1.1–1.9]	**0.005**	1.7 [0.7–3.9]	0.3
**HLA Missmatches**	1.2 [0.97–1.5]	0.08	1.0 [0.7–1.6]	0.8	1.3 [1.0–1.6]	**0.04**	0.7 [0.4–1.1]	0.2
**cold ischemia time (per 5hrs)**	1.6 [1.1–2.2]	**0.007**	0.9 [0.5–1.8]	0.8	1.2 [0.8–1.7]	0.4	0.8 [0.3–2.0]	0.7
**creatinine POD1 (per 1 unit)**	1.05 [0.9–1.2]	0.4			1.1 [0.9–1.2]	0.4		
**creatinine POD2 (per 1 unit)**	1.2 [1.0–1.4]	**0.03**	0.9 [0.7–1.3]	0.6	1.2 [1.0–1.4]	**0.02**	0.9 [0.7–1.2]	0.6
**creatinine POD7 (per 1 unit)**	7.8 [4.1–15.0]	< **0.001**			7.1 [3.7–13.4]	< **0.001**		
**serum NGAL POD1 (per 50 units)**	2.2 [1.5–3.2]	< **0.001**			2.0 [1.4–2.9]	< **0.001**		
**serum NGAL POD2 (per 50 units)**	4.4 [2.8–6.8]	< **0.001**	2.6 [1.4–4.9]	**0.003**	4.0 [2.6–6.2]	< **0.001**	2.6 [1.4–4.8]	**0.003**
**serum NGAL POD7 (per 50 units)**	6.6 [3.6–12.0]	< **0.001**			6.0 [3.3–10.8]	< **0.001**		
**urinary NGAL POD1 (per 50 units)**	1.6 [1.3–1.9]	< **0.001**			1.6 [1.3–1.9]	< **0.001**		
**urinary NGAL POD2 (per 50 units)**	2.4 [1.8–3.2]	< **0.001**	1.7 [1.2–2.5]	**0.003**	2.3 [1.8–3.1]	< **0.001**	1.7 [1.2–2.5]	**0.003**
**urinary NGAL POD7 (per 50 units)**	7.5 [3.8–14.8]	< **0.001**			6.7 [3.5–13.1]	< **0.001**		
**urinary output POD1 (per 100ml)**	0.87 [0.8–0.9]	< **0.001**			0.9 [0.83–0.92]	< **0.001**		
**urinary output POD2 (per 100ml)**	0.88 [0.8–0.9]	< **0.001**	0.9 [0.9–1.0]	**0.001**	0.9 [0.84–0.92]	< **0.001**	0.94 [0.9–1.0]	**0.001**
**urinary output POD7 (per 100ml)**	0.91 [0.87–0.96]	< **0.001**			0.9 [0.87–0.95]	< **0.001**		

OR: odds ratio; HR: hazard ratio; CI: confidence interval; POD: postoperative day; DGF: delayed graft function

**Table 4:** COX regression model to identify predictors for graft loss and death 2 and 5 yrs after KTx.

**Variable**	**multivariate analysis 2 yrs after transplantation**			
	**graft loss**		**death of patient**	
	**HR (95% CI)**	***P***	**HR (95% CI)**	***P***
**Sex f>m**	0.5 (0.1–1.6)	0.2	0.4 (0.1–1.8)	0.2
**recipient age (per 10 years)**	5.2 (2.0–13.7)	**0.001**	5.3 (1.6–17.8)	**0.007**
**donor age (per 10 years)**	0.9 (0.6–1.3)	0.6	0.9 (0.6–1.4)	0.6
**HLA Missmatches**	0.9 (0.6–1.2)	0.4	1.1 (0.7–1.7)	0.7
**cold ischemia time (per 5hrs)**	1.2 (0.6–2.2)	0.6	1.5 (0.7–3.4)	0.3
**creatinine POD2 (per 1 unit)**	1.0 (0.7–1.3)	0.8	1.1 (0.7–1.5)	0.8
**serum NGAL POD2 (per 50 units)**	1.7 (1.0–2.8)	0.056	1.3 (0.7–2.285)	0.5
**urinary NGAL POD2 (per 50 units)**	1.0 (0.8–1.4)	0.8	1.2 (0.9–1.7)	0.3
**Variable**	**multivariate analysis 5 yrs after transplantation**			
	**graft loss**		**death of patient**	
	**HR (95% CI)**	***P***	**HR (95% CI)**	***P***
**Sex f>m**	0.6 [0.2–1.3]	0.19	0.6 [0.2–2.1]	0.38
**recipient age (per 10 years)**	1.5 [0.9–2.4]	0.1	3.7 [1.4–9.6]	**0.01**
**donor age (per 10 years)**	1.1 [0.8–1.5]	0.53	1.0 [0.6–1.6]	0.91
**HLA Missmatches**	1.0 [0.7–1.3]	0.8	1.1 [0.8–1.7]	0.51
**cold ischemia time (per 5hrs)**	1.0 [1.0–1.0]	0.59	1.0 [1.0–1.0]	0.61
**creatinine POD2 (per 1 unit)**	0.9 [0.7–1.1]	0.34	0.9 [0.6–1.2]	0.41
**serum NGAL POD2 (per 50 units)**	1.2 [0.8–1.8]	0.28	1.2 [0.7–1.9]	0.56
**urinary NGAL POD2 (per 50 units)**	1.2 [1.0–1.5]	0.06	1.2 [0.9–1.6]	0.2

HR: hazard ratio; CI: confidence interval; POD: postoperatve day; KTx: kidney transplantation

## Discussion

DGF after kidney transplantation is a persistent problem ranging from 25.5% of deceased-donor [15] to 3–5% of living-donor recipients [16]. In our series, DGF occurred in 30.6% when defining DGF as the need for dialysis in the first week post-transplant [17]. There are various definitions of DGF in literature. Hollmen et al [12] compared the conventional DGF definition with the DGF criteria defined by Halloran [18] with no significant differences in urinary NGAL expression in their patient population. Recently, Moore at al defined DGF as fail of decrease of serum creatinine by 10% on three consecutive days during the first week after transplantation. Median creatinine values in Table 2 also meet these criteria.

Currently, serum creatinine is the most frequently used clinical parameter of post-transplant kidney function although inaccuracy is given due to the impact of age, sex and muscle mass on creatinine generation [19]. Furthermore, the ability of creatinine to detect functional impairment is less than 50% [12]. Therefore, an intense research is ongoing to identify various possible biomarkers in the early postoperative phase that help predicting DGF. Some publications recently revealed NGAL to predict DGF with more or less high accuracy in urine and serum samples post-transplant [12–13, 20–22]. Weakness of most of these studies is low patient number except the work done by Hollmen et al. [12] with a comparable number to the study presented here. We found that urinary and serum NGAL could predict DGF most highly accurate at second postoperative day. These results are similar to studies by Fonseca and Rostami [22–23]. The study by Hollmen [12] and other works on NGAL after kidney transplantation showed that the earliest samples have best prediction power, but not showing NGAL on postoperative day 2. Our data confirm these results with regard to sensitivity analyses in previous studies.

Diuresis, serum creatinine and the need for dialysis are clinical parameters that help to define DGF. Diagnosis might be delayed especially in the early phase after transplantation due to heterogeneous impact factors such as IRI and higher immunosuppressant levels. To improve patient management it is important to individualize patient care as early as possible. To our knowledge, there is no established treatment for DGF. But graft function can be improved by avoiding overdialysis and nephrotoxic agents, for example adjusting the dose and initiation of calcineurin inhibitors [24]. This immunosuppressant monitoring especially during the early posttransplant phase might be a useful tool for improved future graft function. We tried to correlate NGAL with CNI-levels as possible novel tool for immunomonitoring, however not significantly predictive. A CNI-induced increase in NGAL levels could not be observed in our patients in contrast to the description by Cantaluppi et al. [13].

NGAL is expressed in different cells and tissues and plays an important role during IRI [25]. NGAL is produced in the distal nephron [26]. In previous studies, our group demonstrated, that recombinant NGAL, which is administered to NGAL-knockout mice, was uptaken by the proximal tubular cells and can be detected in the urine as proof of filtration from the blood [2]. These findings let us speculate that NGAL is not only a marker of tubular damage but also a filtration marker, but this requires further investigation. NGAL was tracked in both serum and urine compared to several other studies describing only urinary NGAL expression [12, 23, 27–29]. In multivariate analyses we could show, that urinary NGAL as well as serum NGAL can significantly predict DGF on the second postoperative day but not creatinine. These findings are in contrast to a study by Buemi et al [30] that proved only serum NGAL to be a reliable predictor of DGF occurrence but not urinary NGAL. In contrast to Buemi’s study our study population comprised a much higher number of patients (n = 170).

In univariate analyses cold ischemia time was significantly shorter in the non-DGF group. Reduction of ischemia time but also other factors like the use of machine perfusion have been demonstrated beneficial to decrease the rate of DGF [31]. The expression of NGAL in the latter setting could be interesting for future studies of monitoring graft function with sustained perfusion during cold ischemia time.

Using a Cox regression model for graft loss and patient death two and five years after transplantation we identified only recipient age as an independent risk factor. Therefore, strategies to spread the indication of donor organs is strikingly demanded to face the organ shortage and shorten waiting lists like raising the amount of grafts from expanded criteria donors [32]. As described before, NGAL detection is proposed a useful monitoring tool for those settings [12].

Strengths of the study presented here are the appreciable sample size and a long-term follow up of five years. We present a consistently evaluation of urinary NGAL, serum NGAL and urine output from postoperative day 1 to 7 after kidney transplantation as sort of monitoring tool. These clinical data confirm our previous findings of increase in NGAL/Lipocalin-2 levels in terms of acute graft rejection [33]. Major drawback of this study is the heterogeneous patient cohort of unselected donors and recipients.

## Conclusion

DGF remains a problem with high incidence that affects nearly every third patient in our population. As we cannot change patient and recipient age we have to minimize risk factors for developing DGF that can be influenced, e.g. cold ischemia time. Determination of serum and urinary NGAL can help predicting DGF early after kidney transplantation and may improve graft function by close monitoring, fluid management and adaption of immunosuppression in the longrun.

## Supporting information

S1 Table Dataset on 170 kidney transplant patients. (XLSX)

## References

[1] Boros P and Bromberg JS. New cellular and molecular immune pathways in ischemia/reperfusion injury. Am J Transplant 2006;6(4):652–8. doi: 10.1111/j.1600-6143.2005.01228.x 16539620 10.1111/j.1600-6143.2005.01228.x

[2] Aigner F, Maier HT, Schwelberger HG, Wallnoefer EA, Amberger A, Obrist P, et al Lipocalin-2 regulates the inflammatory response during ischemia and reperfusion of the transplanted heart. Am J Transplant. 2007 4;7(4):779–88. doi: 10.1111/j.1600-6143.2006.01723.x 17391123 10.1111/j.1600-6143.2006.01723.x

[3] Kjeldsen L, Cowland JB, Borregaard N. Human neutrophil gelatinase-associated lipocalin and homologous proteins in rat and mouse. Biochim Biophys Acta. 2000 10 18;1482(1–2):272–83. 11058768 10.1016/s0167-4838(00)00152-7

[4] Mishra J, Mori K, Ma Q, Kelly C, Yang J, Mitsnefes M, et al Amelioration of ischemic acute renal injury by neutrophil gelatinase-associated lipocalin. J Am Soc Nephrol. 2004;15:3073–82. doi: 10.1097/01.ASN.0000145013.44578.45 15579510 10.1097/01.ASN.0000145013.44578.45

[5] Mori K and Nakao K. Neutrophil gelatinase-associated lipocalin as the real-time indicator of active kidney damage. Kidney International 2007;71:967–70. doi: 10.1038/sj.ki.5002165 17342180 10.1038/sj.ki.5002165

[6] Boom H, Mallat MJ, de Fijter JW, Zwinderman AH, Paul LC. Delayed graft function influences renal function, but not survival. Kidney Int. 2000 8;58(2):859–66. doi: 10.1046/j.1523-1755.2000.00235.x 10916111 10.1046/j.1523-1755.2000.00235.x

[7] Tomlanovich S, Golbetz H, Perlroth M, Stinson E, Myers BD. Limitations of creatinine in quantifying the severity of cyclosporine-induced chronic nephropathy. Am J Kidney Dis. 1986 11;8(5):332–7. 3538857 10.1016/s0272-6386(86)80107-x

[8] Baboolal K, Jones GA, Janezic A, Griffiths DR, Jurewicz WA. Molecular and structural consequences of early renal allograft injury. Kidney Int. 2002 2;61(2):686–96. doi: 10.1046/j.1523-1755.2002.00149.x 11849412 10.1046/j.1523-1755.2002.00149.x

[9] Siew ED, Ware LB, Ikizler TA. Biological markers of acute kidney injury. J Am Soc Nephrol. 2011 5;22(5):810–20. doi: 10.1681/ASN.2010080796 21493774 10.1681/ASN.2010080796

[10] Hall IE, Yarlagadda SG, Coca SG, Wang Z, Doshi M, Devarajan P, et al IL-18 and urinary NGAL predict dialysis and graft recovery after kidney transplantation. J Am Soc Nephrol. 2010 1;21(1):189–97. doi: 10.1681/ASN.2009030264 19762491 10.1681/ASN.2009030264 PMC2799276

[11] Field M, Lowe D, Cobbold M, Higgins R, Briggs D, Inston N, et al The use of NGAL and IP-10 in the prediction of early acute rejection in highly sensitized patients following HLA-incompatible renal transplantation. Transpl Int. 2014 4;27(4):362–70. doi: 10.1111/tri.12266 24438378 10.1111/tri.12266

[12] Hollmen ME, Kyllönen LE, Inkinen KA, Lalla ML, Salmela KT. Urine neutrophil gelatinase-associated lipocalin is a marker of graft recovery after kidney transplantation. Kidney Int. 2011 1;79(1):89–98. doi: 10.1038/ki.2010.351 20861824 10.1038/ki.2010.351

[13] Cantaluppi V, Dellepiane S, Tamagnone M, Medica D, Figliolini F, Messina M, et al Neutrophil Gelatinase Associated Lipocalin Is an Early and Accurate Biomarker of Graft Function and Tissue Regeneration in Kidney Transplantation from Extended Criteria Donors. PLoS One. 2015 6 30;10(6):e0129279 doi: 10.1371/journal.pone.0129279 eCollection 2015 26125566 10.1371/journal.pone.0129279 PMC4488380

[14] http://www.unos.org.

[15] Huaman MA, Vilchez V, Mei X, Davenport D, Gedaly R. Donor positive blood culture is associated with delayed graft function in kidney transplant recipients: a propensity score analysis of the UNOS database. Clin Transplant. 2016 4;30(4):415–20. doi: 10.1111/ctr.12703 26840885 10.1111/ctr.12703

[16] Redfield RR, Scalea JR, Zens TJ, Muth B, Kaufman DB, Djamali A, et al Predictors and outcomes of delayed graft function after living-donor kidney transplantation. Transpl Int. 2016 1;29(1):81–7. doi: 10.1111/tri.12696 26432507 10.1111/tri.12696

[17] Mallon DH, Summers DM, Bradley JA, Pettigrew GJ. Defining delayed graft function after renal transplantation: simplest is best. Transplantation. 2013 11 27;96(10):885–9. doi: 10.1097/TP.0b013e3182a19348 24056620 10.1097/TP.0b013e3182a19348

[18] Halloran PF, Aprile MA, Farewell V, Ludwin D, Smith EK, Tsai SY, et al Early function as the principal correlate of graft survival. A multivariate analysis of 200 cadaveric renal transplants treated with a protocol incorporating antilymphocyte globulin and cyclosporine. Transplantation. 1988 8;46(2):223–8. 3043779

[19] Slocum JL, Heung M, Pennathur S. Marking renal injury: can we move beyond serum creatinine? Transl Res. 2012 4;159(4):277–89. doi: 10.1016/j.trsl.2012.01.014 22424431 10.1016/j.trsl.2012.01.014 PMC3308350

[20] Reese PP, Hall IE, Weng FL, Schröppel B, Doshi MD, Hasz RD, et al Associations between Deceased-Donor Urine Injury Biomarkers and Kidney Transplant Outcomes. J Am Soc Nephrol. 2016 5;27(5):1534–43. doi: 10.1681/ASN.2015040345 26374609 10.1681/ASN.2015040345 PMC4849827

[21] Parikh CR, Jani A, Mishra J, Ma Q, Kelly C, Barasch J, et al Urine NGAL and IL-18 are predictive biomarkers for delayed graft function following kidney transplantation. Am J Transplant. 2006 7;6(7):1639–45. doi: 10.1111/j.1600-6143.2006.01352.x 16827865 10.1111/j.1600-6143.2006.01352.x

[22] Rostami Z, Nikpoor M, Einollahi B. Urinary Neutrophil Gelatinase Associated Lipocalin (NGAL) for Early Diagnosis of Acute Kidney Injury in Renal Transplant Recipients. ephrourol Mon. 2013 Spring;5(2):745–52. Epub 2013 Mar 30. 23841038 10.5812/numonthly.9385 PMC3703133

[23] Fonseca I, Oliveira JC, Almeida M, Cruz M, Malho A, Martins LS, et al Neutrophil gelatinase-associated lipocalin in kidney transplantation is an early marker of graft dysfunction and is associated with one-year renal function. J Transplant. 2013;2013:650123 doi: 10.1155/2013/650123 24288591 10.1155/2013/650123 PMC3833111

[24] Nashan B, Abbud-Filho M, Citterio F. Prediction, prevention, and management of delayed graft function: where are we now? Clin Transplant. 2016 10;30(10):1198–1208. doi: 10.1111/ctr.12832 27543840 10.1111/ctr.12832

[25] Sickinger S, Maier H, König S, Vallant N, Kofler M, Schumpp P, et al Lipocalin-2 as mediator of chemokine expression and granulocyte infiltration during ischemia and reperfusion. Transpl Int. 2013 7;26(7):761–9. doi: 10.1111/tri.12116 23701109 10.1111/tri.12116

[26] Mishra J, Ma Q, Kelly C, Mitsnefes M, Mori K, Barasch J, et al Kidney NGAL is a novel early marker of acute injury following transplantation. Pediatr Nephrol. 2006 6;21(6):856–63. doi: 10.1007/s00467-006-0055-0 16528543 10.1007/s00467-006-0055-0

[27] Cui LY, Zhu X, Yang S, Zhou JS, Zhang HX, Liu L, et al Prognostic Value of Levels of Urine Neutrophil Gelatinase-associated Lipocalin and Interleukin-18 in Patients With Delayed Graft Function After Kidney Transplantation. Transplant Proc. 2015 12;47(10):2846–51. doi: 10.1016/j.transproceed.2015.10.042 26707300 10.1016/j.transproceed.2015.10.042

[28] Kanter J, Beltran S, Molina D, Vallecillo J, Sancho A, Gavela E, et al Urinary neutrophil gelatinase-associated lipocalin after kidney transplantation: is it a good biomarker to assess delayed graft function? Transplant Proc. 2013 5;45(4):1368–70. doi: 10.1016/j.transproceed.2013.01.019 23726574 10.1016/j.transproceed.2013.01.019

[29] Qurashi S, Ghamdi G, Jaradat M, Tamim H, Aljumah A, Tamimi W, et al Urinary neutrophil gelatinase-associated lipocalin and the occurrence of delayed graft function after kidney transplant. Exp Clin Transplant. 2014 10;12(5):396–400. doi: 10.6002/ect.2013.0300 25019387 10.6002/ect.2013.0300

[30] Buemi A, Musuamba F, Frederic S, Douhet A, De Meyer M, De Pauw L, et al Is plasma and urine neutrophil gelatinase-associated lipocalin (NGAL) determination in donors and recipients predictive of renal function after kidney transplantation? Clin Biochem. 2014 10;47(15):68–72. doi: 10.1016/j.clinbiochem.2014.06.079 25011070 10.1016/j.clinbiochem.2014.06.079

[31] Hameed AM, Pleass HC, Wong G, Hawthorne WJ. Maximizing kidneys for transplantation using machine perfusion: from the past to the future: A comprehensive systematic review and meta-analysis. Medicine (Baltimore). 2016 10;95(40):e5083. 27749583 10.1097/MD.0000000000005083 PMC5059086

[32] United Stated Renal Data System Annual Data Report, 2015. http://www.usrds.org.

[33] Ashraf MI, Schwelberger HG, Brendel KA, Feurle J, Andrassy J, Kotsch K, et al Exogenous Lipocalin 2 Ameliorates Acute Rejection in a Mouse Model of Renal Transplantation. Am J Transplant. 2016 3;16(3):808–20. doi: 10.1111/ajt.13521 Epub 2015 Nov 23. 26595644 10.1111/ajt.13521 PMC4996417

